# Knowledge, attitude, and practice toward advanced precision radiotherapy among patients with head and neck cancer

**DOI:** 10.3389/fpubh.2024.1461808

**Published:** 2024-10-17

**Authors:** Zhanfei Guo, Qingrui Cai, Bingbing Liu, Liufang Zhao, Yanyan Xie, Zhijia Li, Rui Liu, Yunxiao Wang, Xiaodan Chen, Zhiwei Zhang

**Affiliations:** ^1^College of Clinical Medicine, Hebei University of Engineering, Handan, China; ^2^Department of Oncology, Affiliated Hospital of Hebei University of Engineering, Handan, China; ^3^Department of Respiratory and Critical Care Medicine, Affiliated Hospital of Hebei University of Engineering, Handan, China; ^4^First Department of Head and Neck Surgery, Yunnan Cancer Hospital, Kunming, China; ^5^Department of Medical Oncology, The People's Hospital of Guangxi Zhuang Autonomous Region, Nanning, China

**Keywords:** knowledge, attitude, practice, precision radiotherapy, head and neck cancer

## Abstract

**Background:**

Advancements in radiotherapy (RT) technology have led to the prominence of precision RT in head and neck cancer (HNC) treatment. The new progress in precision RT offers more efficient therapy, potentially improving outcomes for HNC patients.

**Objective:**

The present cross-sectional study aimed to assess the knowledge, attitude, and practice (KAP) of patients in advanced precision RT for HNC treatment.

**Methods:**

This study enrolled HNC patients at the Affiliated Hospital of Hebei University of Engineering between October 2023 and May 2024. Then, the demographic data and KAP scores were collected using an investigator-designed questionnaire. Afterwards, descriptive statistics were provided for all study variables, and the relationship among KAP was analyzed using appropriate statistical tests, including Spearman correlation, logistic regression, and path analysis.

**Results:**

A total of 436 participants with a mean age of 52.03 ± 12.19 years old were included. The mean knowledge score, attitude score, and practice score were 18.33 ± 4.21, 36.14 ± 1.71, and 26.26 ± 1.83, respectively. Although most of the participants were unfamiliar with advanced precision RT, they expressed a high willingness to follow their doctor's recommendation for this treatment. The multivariable analysis revealed a positive association between attitude score and proactive practice. The path analysis revealed that knowledge directly influenced attitude and practice, while attitude directly impacted practice.

**Conclusion:**

HNC participants had poor knowledge of advanced precision RT techniques, but had a positive attitude and the willingness to undergo treatment when recommended by their physicians. These results suggest that improving patients' awareness for advanced precision RT can help to promote better attitude and advanced precision RT practice.

## 1 Introduction

Head and neck cancer (HNC) encompasses malignancies of the oral cavity, lips, pharynx (nasopharynx, oropharynx, and hypopharynx), larynx (glottic, supraglottic and subglottic larynx), ethmoid sinus, maxillary sinus, and salivary glands, mucosal melanoma, and other rare cancers (1). In 2022, approximately 946,477 new cases of the lip, oral, laryngeal, nasopharyngeal, oropharyngeal, hypopharyngeal, and salivary gland cancers (4.7% of all cancers) were diagnosed, with 481,941 deaths (4.9% of all cancer-related deaths) attributed to the disease (2). Globally, the incidence of HNC is increasing, posing a significant public health burden (3, 4).

Management of HNC typically involves a multidisciplinary approach and includes surgery, systemic therapies, radiotherapy (RT), and other interventions. However, these treatments are frequently associated to adverse effects, particularly impacting nutrition and causing oral mucositis (5–7). Surgery may lead to swallowing difficulties, while RT and chemotherapy would commonly cause oral mucositis, taste alterations, decreased appetite, and other nutritional challenges, all of which disrupt the patients' normal eating patterns and nutritional intake (8, 9). Given these treatment-related adverse effects, improving interventions to enhance the patients' quality of life and treatment outcomes has become critical (10, 11). Traditional precision RT regimens, such as three-dimensional conformal radiotherapy (3D-CRT) or intensity-modulated radiotherapy (IMRT), often yield variable responses and severe adverse effects in the treatment of HNC, highlighting the limitations of these approaches.

In recent years, the continuous development of medical science and technology has led to the emergence of advanced precision RTs that utilize sophisticated imaging technology and computer technologies. These methods, such as TomoTherapy (TOMO therapy), CyberKnife, and proton therapy (PT), have become efficient, individualized, and accurate cancer treatment options, ushering in a new era of HNC treatment. Precision TOMO therapy, which is a spiral computed tomography (CT) radiation therapy system, offers significant advantages over traditional 3D-CRT and IMRT by delivering more accurate and effective treatment through rotating CT scans and multi-directional beams (12, 13). In addition, CyberKnife, a type of stereotactic body radiation therapy (SBRT), minimizes damage to normal tissue through highly precise dose delivery (14, 15). PT, which is another advanced precision particle RT, features beams that exponentially decline with increasing tissue depth, depositing most of the energy deep in the tumor tissue while causing minimal damage to surrounding normal tissues (16, 17). Advanced precision RT represents a crucial step toward achieving optimal outcomes (18). These approaches, which are grounded in scientific evidence, can help prevent or minimize adverse effects on function, maintaining the nutritional status, enhancing immune function, shortening hospital stays, and ultimately improving quality of life.

Proper knowledge of the treatment options and expectations for efficacy and side effects may help patients make informed decisions (19, 20). Knowledge, attitude and practice (KAP) studies provide quantitative and qualitative data by identifying misconceptions and misunderstandings that can act as barriers to the optimal treatment implementation within a specific population (21). These studies can inform discussions between patients and physicians, by highlighting areas where patients may require additional knowledge or clarification. Previous KAP studies on RT of HNC mainly focus on complications and dental care (22, 23). Although, studies have shown positive perceptions of TOMO therapy in breast cancer patients (24), the KAP of HNC patients on advanced precision RT remains unclear.

The present study aims to bridge the present gap by preliminarily investigating KAP for advanced precision RT in patients with HNC. The present findings suggest a relatively low level of patient knowledge on advanced precision RT. These study results may inform the introduction of new advanced precision RT technologies into clinical practice, potentially benefiting a greater number of patients with HNC.

## 2 Materials and methods

### 2.1 Study design and participants

This cross-sectional study enrolled HNC patients at the Affiliated Hospital of Hebei University of Engineering from October 2023 to May 2024. The inclusion criteria were as follows: (1) ≥ 18 years old, (2) diagnosis of malignant HNC confirmed by histology or cytology, and (3) Eastern Cooperative Oncology Group performance status ≤ 2. The exclusion criteria were as follows: (1) diagnosis of other malignant tumors within the past 5 years, except for cured cases of basal cell carcinoma or squamous cell carcinoma of the skin, superficial bladder cancer, cervical intraepithelial neoplasia, or ductal carcinoma *in situ* of the breast treated with local treatment, (2) patients with communication disorders, and (3) patients with psychiatric disorders. This study was approved by the Medical Ethics Committee of the Affiliated Hospital of Hebei University of Engineering (Approval No: 2023[K]103, Date: October 18, 2023). All the participants signed the written informed consent after fully understanding the study's objectives, who were voluntary to take part in the study.

### 2.2 Questionnaire

The questionnaire design was based on NCCN Guidelines Insights: Head and Neck Cancers (Version 1.2022) (1); Radiation-induced oral mucositis: A review of current literature on prevention and management (25); Advancements of RT for recurrent head and neck cancer in the modern era (26). After the first draft was completed, a panel of four oncology specialists was consulted. Each expert, with over 10 years of experience, rigorously reviewed the questionnaire items to ensure their accuracy and relevance, leading to the removal of any items deemed incorrect or inappropriate, thereby enhancing the content validity. The questionnaire was revised based on their feedback. A preliminary survey of 25 participants was conducted, yielding a Cronbach's α value of 0.820, which indicated good internal consistency. Therefore, after the pilot study, the questionnaire was improved and all the questions were made more clearly.

The final questionnaire was in Chinese and comprised four sections:

- Demographics: age, gender, education level, weight, height, lifestyle habits, dietary status, body mass index (BMI) and present treatment plans. BMI was calculated as BMI=weight (kg)/height (m)^2^.
- Knowledge: this consisted of 20 items. The responses were scored using a three-point scale: 2-point for “very familiar,” 1-point for “heard of,” and 0-point for “unclear.” The total scores ranged within 0-40 points.
- Attitude: this consisted of eight questions. A 5-point Likert scale was used, which ranged from “strongly agree” to “strongly disagree”. The score ranged from 8 to 40 points.
- Practice: this consisted of seven items. The responses for items P1-P6 responses ranged from “never” to “always”, and were scored from 1 to 5. The total score ranged within 6–30 points. Since item P7 did not show positive or negative attitude tendencies, this was descriptively analyzed. Scores that exceeded 70% of the maximum possible mark in each section were considered indicative of good knowledge, a positive attitude, and proactive practice (27).

### 2.3 Questionnaire distribution and quality control

The questionnaires were distributed to the study participants offline in the hospital wards and collected on the spot. All questionnaires were collected anonymously. Research assistants received thorough training on the entire process, such as elucidating the questionnaire's contents to participants, managing the distribution and collection of questionnaires, rules for filling questionnaires, and procedures for data input. In the knowledge section, the participants should answer the knowledge questions directly and avoided other factors influencing their genuine responses. In the attitude and practice section, our research assistants distributed an educational brochure introducing advanced precision RT, and clarified any doubts or questions the participants had. This approach helped them gain a basic understanding of advanced precision RT and maintained the integrity of the data collection process. One member of the research team was responsible for the distribution and collection of questionnaires, and then another researcher input the data into the database and verified the results for accuracy.

### 2.4 Statistical analysis

The study sample size was determined according to a previous study (28) using the following formula:


$$\begin{array}{l} n=\frac{z^{2}pq}{e^{2}} \end{array}$$

Where “*n*” refers to the number of participants, “z” equals to 1.96 (which corresponds to a 95% confidence interval), “*p*” refers to the anticipated proportion, “*q*” equals to 1-*p*, and “e” refers to the margin of error (which is fixed at 5%). A conservative approach was adopted, and 50% was selected as the expected proportion to optimize the sample size. This calculation yielded a required sample size of 384. In order to account for potential participant loss, a theoretical sample size of 422 was targeted (including a 10% buffer).

The data analysis was performed using SPSS 26.0 and AMOS 24.0 (IBM, Armonk, NY, USA). The normality of continuous data was assessed using the Kolmogorov-Smirnov test. The continuous variables that conformed to the normal distribution were presented in means ± standard deviations (SD) and analyzed using Student's *t*-test (two groups) or ANOVA (more than two groups). Data with a skewed distribution were presented in medians (range) and analyzed by the Wilcoxon-Mann-Whitney U-test (two groups) or the Kruskal-Wallis analysis of variance (more than two groups). Categorical variables were presented in n (%) and analyzed by the chi-squared test. Spearman correlation coefficients were used to analyze the correlations among the KAP dimensions. Univariable and multivariable logistic regression were conducted to identify the independent factors associated to proactive practice [defined as a score that exceeded 70% of the maximum possible score (27)]. Variables with *p* < 0.05 in the univariable analyses were included in the multivariable analysis. Path analysis was employed to test the following hypotheses: (H1) knowledge directly affects attitude; (H2) knowledge directly affects practice; (H3) knowledge indirectly affects practice through attitude. Two-sided *p* < 0.05 were considered statistically significant.

## 3 Results

### 3.1 Characteristics of the participants

**Figure 1:**
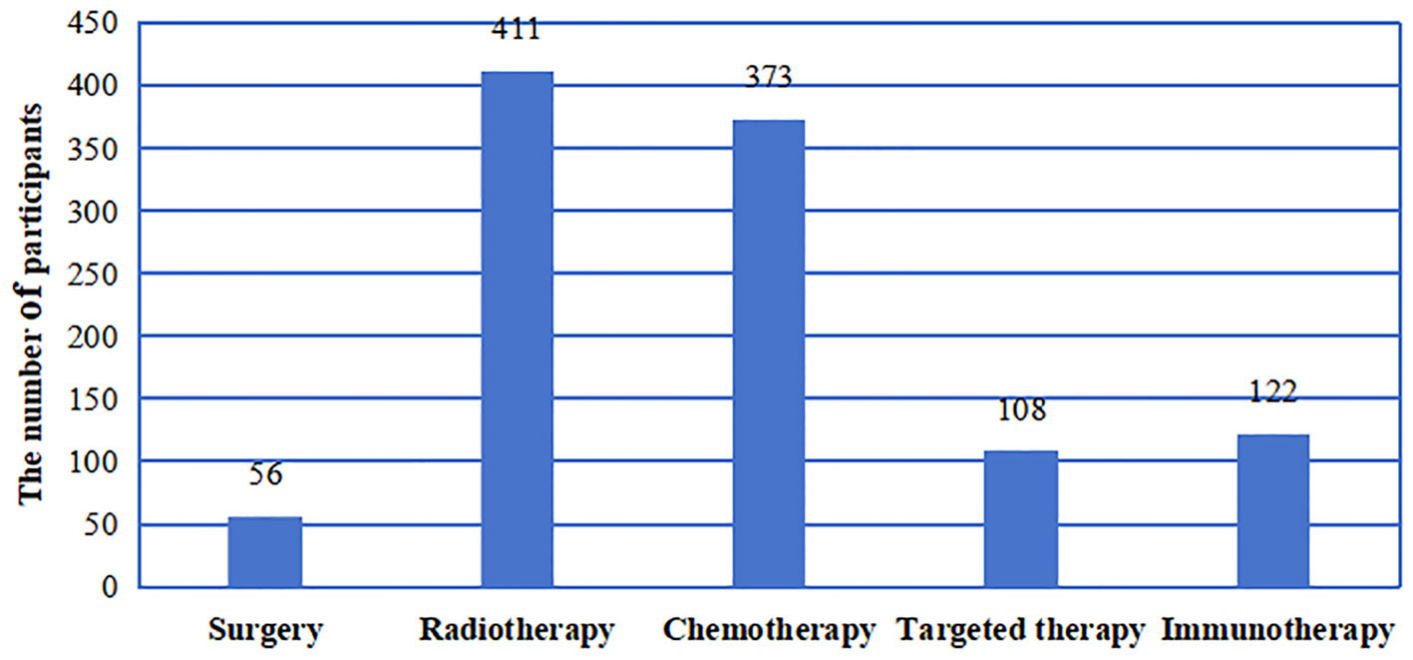
The number of the patients with HNC received various treatments were analyzed.

A total of 440 questionnaires were collected. Among these questionnaires, one questionnaire had abnormal data, and three were incomplete. Thus, a total of 436 valid questionnaires were analyzed. Among all the participants, 339 (77.75%) were male, and 97 (22.25%) were female. The average participant age was 52.03 (± 12.19) years old with a mean BMI of 22.84 (± 2.88) kg/m^2^. Half of the participants reported smoking (50.00%). The characteristics of the participants were as follow (Table 1): rural residence (57.11%), primary school education or below (33.03%), monthly income of 2,000–5,000 CNY (60.78%), drinking (56.88%), not using betel nut (95.64%), reducing dietary intake (60.55%), diagnosed with nasopharyngeal cancer (75.23%), not undergoing advanced precision RT (97.71%), and experiencing treatment-related complications (95.64%). The other detail information was shown in the Table 1. Among the 436 participants, 12.84% of the participants underwent surgery, 94.27% of the participants received RT, 85.55% received chemotherapy, 24.77% received targeted therapy, and 27.98% received immunotherapy (Figure 1).

**Table 1:** Characteristics of the participants.

**Variables**	***N* (%)**	**Knowledge score**		**Attitude score**		**Practice score**	
		**Mean** ±**SD**	***p***	**Mean** ±**SD**	***p***	**Mean** ±**SD**	***p***
**Total**	436	18.33 ± 4.21		36.14 ± 1.71		26.26 ± 1.83	
**Age**			< 0.001		0.033		0.214
18–40	81 (18.58)	18.47 ± 4.43		36.19 ± 1.90		26.37 ± 1.74	
41–60	250 (57.34)	18.87 ± 4.19		36.26 ± 1.64		26.39 ± 1.68	
>60	105 (24.08)	16.95 ± 3.81		35.83 ± 1.68		25.88 ± 2.16	
**Gender**			0.613		0.680		0.240
male	339 (77.75)	18.40 ± 4.27		36.13 ± 1.71		26.22 ± 1.83	
female	97 (22.25)	18.09 ± 4.00		36.20 ± 1.71		26.43 ± 1.82	
**Residence**			< 0.001		0.294		0.936
Urban	187 (42.89)	19.53 ± 4.21		36.21 ± 1.74		26.30 ± 1.79	
Rural	249 (57.11)	17.43 ± 3.99		36.09 ± 1.68		26.23 ± 1.86	
**Education**			< 0.001		0.022		0.703
Primary school and below	144 (33.03)	17.00 ± 3.50		36.04 ± 1.50		26.20 ± 1.74	
Junior high school	102 (23.39)	17.71 ± 4.16		36.01 ± 1.86		26.32 ± 2.01	
High school/technical school	77 (17.66)	18.88 ± 4.39		35.86 ± 2.11		26.17 ± 1.79	
College	80 (18.35)	19.60 ± 3.65		36.49 ± 1.39		26.26 ± 1.79	
Bachelor's degree and above	33 (7.57)	21.73 ± 5.22		36.85 ± 1.48		26.58 ± 1.82	
**Average monthly income (CNY)**			< 0.001		0.071		0.750
< 2,000	79 (18.12)	16.99 ± 3.83		35.84 ± 1.90		26.15 ± 2.12	
2,000–5,000	265 (60.78)	18.14 ± 3.96		36.14 ± 1.69		26.31 ± 1.79	
>5,000	92 (21.10)	20.04 ± 4.69		36.42 ± 1.55		26.22 ± 1.66	
**Smoking**			0.117		0.532		0.840
Yes	218 (50.00)	17.99 ± 4.10		36.06 ± 1.79		26.22 ± 2.00	
No	218 (50.00)	18.68 ± 4.30		36.22 ± 1.62		26.31 ± 1.64	
**Alcohol consumption**			0.055		0.325		0.512
Yes	248 (56.88)	18.02 ± 4.16		36.06 ± 1.75		26.21 ± 1.86	
No	188 (43.12)	18.75 ± 4.26		36.26 ± 1.64		26.33 ± 1.78	
**Daily betel nut chewing**			0.779		0.921		0.421
Yes	19 (4.36)	17.79 ± 3.21		36.16 ± 1.30		26.05 ± 1.27	
No	417 (95.64)	18.36 ± 4.25		36.14 ± 1.72		26.27 ± 1.85	
**Current dietary status**			0.565		0.036		0.365
Normal intake	172 (39.45)	18.55 ± 4.49		36.22 ± 1.80		26.37 ± 1.69	
Slightly reduced intake	211 (48.39)	18.11 ± 4.01		36.21 ± 1.47		26.12 ± 1.89	
Intake reduced by half	49 (11.24)	18.71 ± 3.94		35.96 ± 1.73		26.61 ± 1.84	
Almost no intake	4 (0.92)	16.25 ± 5.85		31.75 ± 3.50		25.25 ± 3.50	
**Tumor type**			0.005		0.011		0.761
Oral cancer	10 (2.29)	14.30 ± 3.95		34.10 ± 3.57		25.40 ± 3.34	
Oropharyngeal cancer	31 (7.11)	17.26 ± 4.19		35.87 ± 1.80		26.29 ± 1.94	
Laryngeal cancer	19 (4.36)	18.26 ± 2.84		35.84 ± 1.74		25.74 ± 2.33	
Hypopharyngeal cancer	17 (3.90)	17.59 ± 2.24		35.94 ± 1.03		25.94 ± 1.68	
Nasopharyngeal cancer	328 (75.23)	18.52 ± 4.24		36.25 ± 1.63		26.31 ± 1.76	
Primary unknown squamous cell carcinoma of the neck lymph nodes	1 (0.23)	17.00 ± 0.00		36.00 ± 0.00		26.00 ± 0.00	
Salivary gland cancer	9 (2.06)	23.11 ± 6.45		37.44 ± 0.88		27.11 ± 1.45	
Other tumors	21 (4.82)	17.52 ± 2.94		35.76 ± 1.48		26.33 ± 1.43	
**Underwent advanced precision RT**			0.364		0.837		0.089
Yes	10 (2.29)	20.80 ± 7.80		35.60 ± 3.53		27.10 ± 2.92	
No	426 (97.71)	18.27 ± 4.09		36.16 ± 1.65		26.24 ± 1.79	
**Treatment-related complications**			0.001		< 0.001		0.072
Yes	417 (95.64)	18.50 ± 4.13		36.22 ± 1.64		26.33 ± 1.73	
No	19 (4.36)	14.63 ± 4.34		34.58 ± 2.36		24.89 ± 3.11	

### 3.2 Knowledge of precision RT in HNC patients

The average knowledge score for precision RT in HNC (Table 1) was 18.33 ± 4.21 (range: 0–40). The average knowledge score per age group was shown as follows: 18–40 years old had an average knowledge score 18.47 ± 4.43, 41–60 years old had an average knowledge score 18.87 ± 4.19, and >60 years old had an average knowledge score 16.95 ± 3.81. Specifically, the results indicated significant differences in knowledge (*p* < 0.001) and attitude (*p* = 0.033) across age groups, though no significant difference was observed in practice scores (*p* = 0.214). Regarding gender, there were no significant differences in knowledge (*p* = 0.613), attitude (*p* = 0.680), or practice (*p* = 0.240) scores between male and female participants (Table 1). Residence, education, income, HNC type, using advanced new techniques for precision RT and treatment complications were significantly associated with knowledge scores. Knowledge dimension distribution was as follows (Table 2): The knowledge item with the highest score was K19 (99.77%; “During treatment, one should consume foods rich in minerals and vitamins, such as leafy vegetables and fruits”). Conversely, the lowest score was K10 (5.73%; “PT, CyberKnife, and other advanced radiotherapies are presently the best radiotherapy technologies, which can strictly limit the radiation dose strictly to the lesion area, reducing radiation exposure to adjacent organs and normal tissues”). Most of the participants had a general lack of awareness on specific technologies, such as helical TOMO therapy (item K9; 93.12%) or CyberKnife, PT and other new precision RT technologies (item K10; 94.27%).

**Table 2:** Knowledge dimension distribution.

**Variables**	**Very familiar *n* (%)**	**Heard of *n* (%)**	**Not clear *n* (%)**
1. Head and neck tumors mainly include tumors of the oral and maxillofacial region, ear, nose, and throat, as well as neck tumors.	5 (1.15)	167 (38.3)	264 (60.55)
2. Head and neck tumors are one of the most common tumors that lead to malnutrition in patients.	17 (3.9)	191 (43.81)	228 (52.29)
3. Risk factors for head and neck tumors include smoking, alcohol consumption, poor oral hygiene, long-term betel nut chewing, human papillomavirus (HPV) infection, and genetic factors.	273 (62.61)	145 (33.26)	18 (4.13)
4. Gene mutations are one of the causes of head and neck tumors.	4 (0.92)	122 (27.98)	310 (71.1)
5. Second-generation gene sequencing can detect gene mutation sites, providing precise diagnosis for head and neck tumors. It can also help select targeted and immunotherapeutic drugs that are most likely to benefit patients.	5 (1.15)	108 (24.77)	323 (74.08)
6. Pathological biopsy, HPV detection, EB virus detection, and detection of immune-related markers are also diagnostic methods for head and neck tumors.	277 (63.53)	143 (32.8)	16 (3.67)
7. Treatment options for head and neck tumors vary, mainly depending on the stage and type of cancer.	26 (5.96)	324 (74.31)	86 (19.72)
8. Treatment options for head and neck tumors include surgery, radiotherapy, chemotherapy, targeted therapy, and immunotherapy.	47 (10.78)	374 (85.78)	15 (3.44)
9. TOMO helical tomotherapy is more precise, can improve the survival rate of patients with head and neck tumors, and reduces the incidence of complications.	5 (1.15)	25 (5.73)	406 (93.12)
10. Proton RT, CyberKnife etc. and other advanced radiotherapy are currently the best radiotherapy technologies, which can limit the radiation dose strictly to the lesion area, reducing radiation exposure to adjacent organs and normal tissues.	6 (1.38)	19 (4.36)	411 (94.27)
11. Patients with head and neck tumors should pay attention to maintaining oral and oropharyngeal hygiene, rinsing the mouth frequently, and drinking plenty of water to keep the mucous membranes moist. Soft and liquid foods should be consumed mainly to avoid damaging the oral mucosa by consuming oropharyngeal hygiene hot foods or liquids	253 (58.03)	171 (39.22)	12 (2.75)
12. Common complications during treatment include erythema on the skin, pain or burning sensation on the skin, dry mouth, loss of taste, hoarseness or difficulty swallowing.	278 (63.76)	143 (32.8)	15 (3.44)
13. Targeted therapy uses targeted drugs to identify tumor cells and kill them precisely. The latest drugs include cetuximab, nivolumab, etc.	20 (4.59)	187 (42.89)	229 (52.52)
14. Immunotherapy can enhance the effectiveness of other cancer treatment methods.	21 (4.82)	157 (36.01)	258 (59.17)
15. A combination of targeted therapy and immunotherapy may be a new treatment option for patients intolerant to chemotherapy.	16 (3.67)	146 (33.49)	274 (62.84)
16. Treatment complications can affect the nutritional status of patients, potentially leading to malnutrition.	131 (30.05)	267 (61.24)	38 (8.72)
17. Nutritional supplementation therapy during radiotherapy can effectively alleviate complications.	214 (49.08)	185 (42.43)	37 (8.49)
18. During treatment, it is advisable to consume foods rich in protein, such as fish, poultry, and eggs.	260 (59.63)	176 (40.37)	0
19. During treatment, one should consume foods rich in minerals and vitamins, such as leafy vegetables and fruits.	288 (66.06)	147 (33.72)	1 (0.23)
20. Intake of refined sugars, salt, and pickled foods should be limited.	110 (25.23)	274 (62.84)	52 (11.93)

### 3.3 Attitude toward precision RT in HNC

The average attitude score (Table 1) was 36.14 ± 1.71 (range: 8–40). Differences in attitude scores were identified based on education, dietary status, HNC type, us of advanced and new techniques for precision RT, and complications. The attitude dimension distribution was as follows (Supplementary Table S1): The attitude item with the highest score was A6 (99.54%; “I believe that strict maintenance of oral hygiene is crucial and can effectively prevent the occurrence of treatment-related oral complications”), while the item with the lowest score was A2 (86.69%; “If recommended by my doctor, I am willing to undergo genetic sequencing tests”). Most of the participants expressed their willingness to undergo RT (item A3, 97.25%).

### 3.4 Practices toward precision RT of HNC

The average practice score (Table 1) was 26.26 ± 1.83 (range: 6–30). No significant differences in practice scores was observed across the participant characteristics. The practice dimension distribution was as follow (Supplementary Table S2): The practice item with the highest score was P5 (99.31%; “I strictly abstain from smoking and alcohol consumption during treatment.”), while the item with the lowest score was P3 (49.31%; “I actively consult with doctors on dietary nutrition and scientific nutrition.”).

### 3.5 The main means for participants to obtain information on precision RT

**Figure 2:**
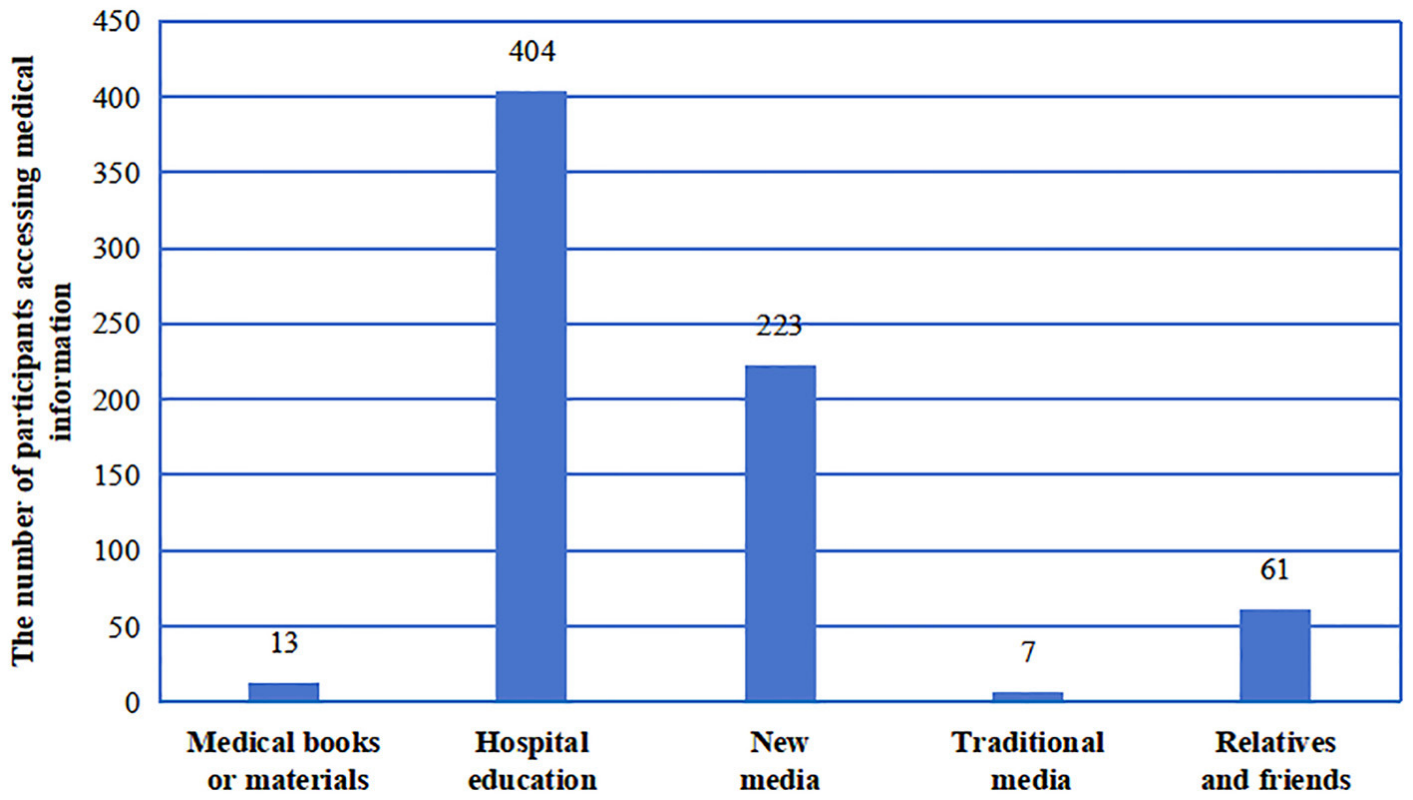
The different sources of knowledge about advanced precision radiotherapy were obtained by HNC patients.

The primary sources of information on cancer treatment and complications contained hospital education (92.66%), followed by social media (51.15%), relatives and friends (13.99%), medical books or materials (2.98%), and traditional media (1.61%) (Figure 2). This highlights the diverse avenues through which individuals access medical information in the digital age, suggesting that medical professionals can leverage new media to disseminate knowledge on precision RT technology to the public.

### 3.6 Correlation analysis of knowledge, attitude, and practice

The correlation analysis (Table 3) revealed significant positive correlations between knowledge scores and attitude scores (r = 0.309, *p* < 0.001), and between knowledge scores and practice scores (r = 0.231, *p* < 0.001). In addition, there was a significant positive correlation between the attitude scores and practice scores (r = 0.174, *p* < 0.001). These results indicated that higher levels of knowledge on precision RT are associated to more positive attitudes, and these positive attitudes further enhance proactive practices among patients.

**Table 3:** Correlation analysis among knowledge, attitude, and practice.

	**Knowledge**	**Attitude**	**Practice**
Knowledge	1		
Attitude	0.309 (*P* < 0.001)	1	
Practice	0.231 (*P* < 0.001)	0.174 (*P* < 0.001)	1

### 3.7 Univariable and multivariable logistics regression analysis for practice

The factors that influenced practice were evaluated by univariable and multivariable logistics regression analysis, in order to identify the possible predictors that affect practice toward advanced precision RT (Table 4). Univariate analysis revealed that knowledge score (OR = 1.091, 95% CI: 1.013–1.176, *p* = 0.021), attitude score (OR = 1.319, 95%CI: 1.135–1.534, *p* < 0.001), BMI (OR = 1.120, 95% CI: 1.010-1.242, *p* = 0.031), almost no intake (OR = 0.110, 95% CI: 0.015–0.829, *p* = 0.032), with treatment-related complications (OR = 3.815, 95% CI: 1.382–10.534, *p* = 0.010), and RT experience (OR = 3.244, 95% CI: 1.283–8.199, *p* = 0.013) were significantly associated to practice scores. Multivariate analysis was performed when the *p*-value was < 0.05 in univariate analysis. However, in the multivariable analysis, merely the only attitude scores (OR = 1.214, 95%CI: 1.019–1.447, *p* = 0.030) remained as the an independent influence factor. These findings suggested that although knowledge levels and certain clinical factors influence practice to some extent, attitudes plays a more substantial role in determining a patient' proactive behavior.

**Table 4:** Univariable and multivariable analyses of the practice.

**Variable**	**Univariable logistic regression**		**Multivariable logistic regression**	
	**OR (95%CI)**	***P***	**OR (95%CI)**	***P***
Knowledge score	1.091 (1.013–1.176)	0.021	1.040 (0.960–1.128)	0.336
Attitude score	1.319 (1.135–1.534)	< 0.001	1.214 (1.019–1.447)	0.030
**Age (years)**				
18-40	1.478 (0.824–2.650)	0.190	-	-
41-60	1.228 (0.774–1.949)	0.383	-	-
>60	ref	-	-	-
**Gender**		0.121		
Male	0.699 (0.444–1.099)	-	-	-
Female	ref	-	-	-
Body mass index	1.120 (1.010–1.242)	0.031	1.082 (0.972–1.204)	0.151
**Current dietary status**				
Normal intake	ref	-	-	-
Slightly reduced intake	0.780 (0.408–1.491)	0.453	-	-
Intake reduced by half	0.786 (0.292–2.116)	0.634	-	-
Almost no intake	0.110 (0.015–0.829)	0.032	0.501 (0.047–5.370)	0.568
**Treatment-related complications**				
Yes	3.815 (1.382–10.534)	0.010	1.590 (0.340–7.427)	0.555
No	ref	-	-	-
**Radiotherapy**				
Yes	3.244 (1.283–8.199)	0.013	1.848 (0.473–7.220)	0.377
No	ref	-	-	-

### 3.8 Path analysis

**Figure 3:**
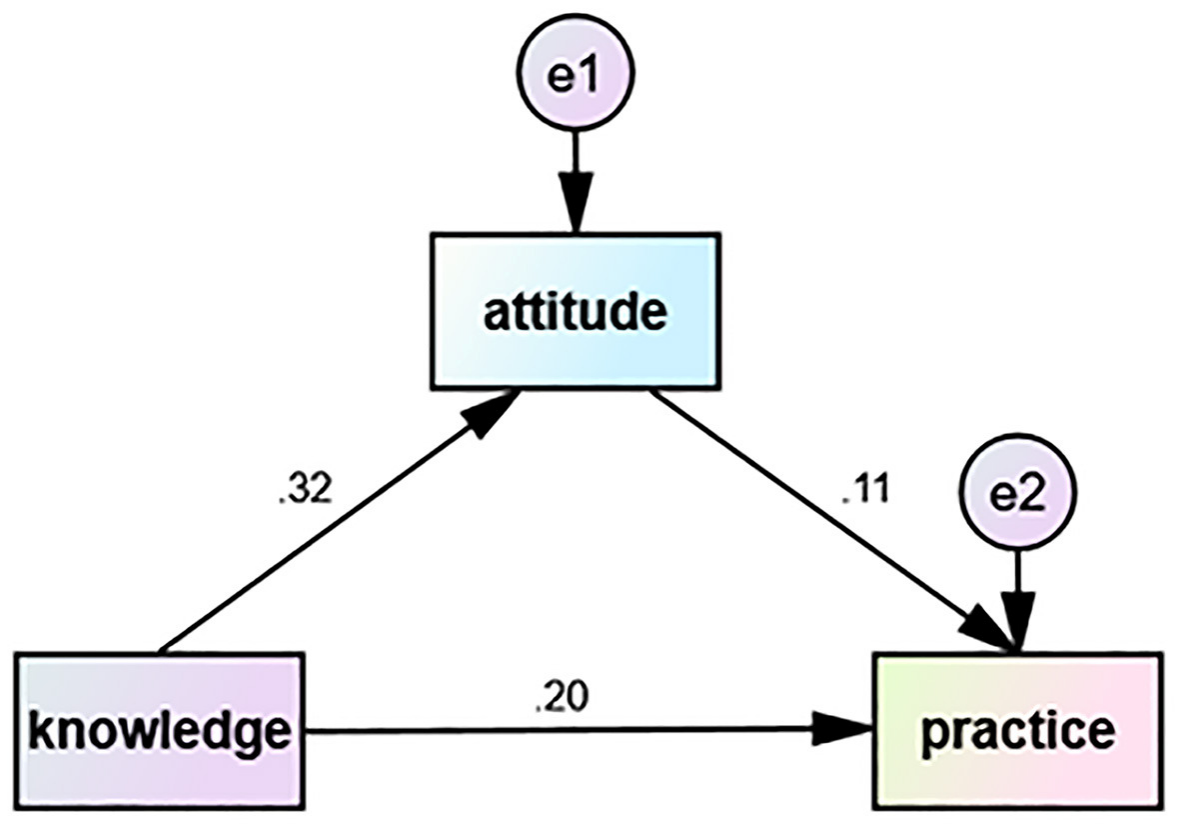
The correlation between knowledge, attitude, and practice scores for advanced precision radiotherapy in HNC was analyzed using path analysis. The direction of causality is displayed by a single-headed arrow.

The path analysis (Table 5, Figure 3) further revealed the complex relationships among KAP. The results revealed that knowledge directly influenced both attitude (β = 0.131, *p* < 0.001) and practice (β = 0.087, *p* < 0.001), and that attitudes directly influenced practice (β = 0.121, *p* = 0.021). This finding indicates that knowledge impacts practice not only directly but also indirectly by improving attitude. These findings highlight the importance of enhancing both knowledge and attitudes through patient education, in order to foster proactive practice in precision RT.

**Table 5:** Path analysis of knowledge, attitude, and practice.

**Path**	**β**	**Standardized β**	**S.E**.	**C.R**.	***P***
Attitude < – Knowledge	0.131	0.323	0.018	7.115	< 0.001
Practice < – Knowledge	0.087	0.201	0.021	4.103	< 0.001
Practice < – Attitude	0.121	0.113	0.052	2.317	0.021

## 4 Discussion

Advancements in RT technology have introduced innovative approaches. The present study contributes to the growing body of evidence supporting the clinical adoption of advanced precision RT technologies (TOMO therapy, CyberKnife, PT, etc.), which outperform traditional precision RT. The present datas suggest that although HNC patients generally lacked knowledge on advanced precision RT, they demonstrated a positive attitude and a willingness to participate in the treatment. In addition, significant positive correlations were observed the KAP scores. These findings underscored the critical role of patient education and communication in optimizing HNC treatment.

The knowledge of new advanced precision RT technologies is very important for HNC patients, which help the patients to make a better treatment option. Possessing a strong foundation of knowledge on available treatment options can help patients make the choice when discussing with their oncologists, and develop realistic expectations for treatment outcomes (19, 20). The results of this study revealed poor knowledge of advanced precision RT technologies among patients. Most of studies are consistent with our findings. For example, a survey on patients' knowledge of PT among oncology patients reported a mean knowledge score of 3.4 ± 3.6 (range: 0–12), indicating insufficient knowledge (29). Another study investigating KAP in rectal cancer patients regarding chemoradiotherapy similarly found limited knowledge (30). In addition, a study conducted in Tanzania revealed that the public had poor knowledge of RT, with an average correct response rate of 35.6% across 13 awareness items (31). Thus, the participants always had low level of knowledge score in the KAP studies, which indicated that there was a need to improve the knowledge.

The study also found that rural residents, participants with lower educational levels, and those with lower incomes had lower knowledge scores. Rural populations often have limited access to healthcare resources and educational materials, which restricts their health literacy. Studies have consistently reported that geographic disparities, particularly between rural and urban areas, affect the availability and quality of healthcare information, leading to poorer health outcomes in rural communities (32). Lower educational attainment has also been strongly correlated with reduced health literacy, as individuals with less education may struggle to comprehend complex medical information and treatment options, resulting in a lower understanding of advanced medical technologies like RT (33, 34). Similarly, low-income individuals face additional barriers such as limited access to healthcare services, financial constraints, and less frequent interactions with healthcare providers, which contribute to a gap in knowledge (35). These findings emphasize the need for tailored educational interventions that address the specific challenges of rural, low-income, and less-educated populations, which are essential for improving health outcomes and ensuring equitable access to information on advanced treatments.

The findings showed that participants exhibited low knowledge but maintained a positive attitude toward advanced precision RT, and the similar results were also seen in other studies (36, 37). A study on rectal cancer patients undergoing chemoradiotherapy is consistent with our research, they also found that limited knowledge was coexisted with favorable attitude (30). Another study found that patients often showed positive attitude after consulting the suggestions of healthcare providers, although they did not know much about the new knowledge or technology (38). When faced with complex medical decisions, such as the use of advanced precision RT, patients may rely on the expertise of their doctors rather than seeking to fully understand the intricacies of the treatment (39). Additionally, patients' positive attitude could be driven by the perception that advanced technologies are superior and offer better outcomes, despite their limited knowledge of the specific techniques. Another possible reason might be the lower incidence of HNC compared to common conditions like hypertension or diabetes, which leads to less public propaganda and lower awareness (40). However, given the severe impact of HNC on longevity and quality of life, participants may have stronger expectations for better treatments, fostering a positive attitude despite limited knowledge.

Studies have reported that a higher socioeconomic status is generally associated to better health literacy (41), which translates into more active engagement in medical consultations (42). However, in the present study, the knowledge scores or the socioeconomic characteristics were not independently associated to the practice scores. However, attitude scores had an independent association to the practice. A similar finding was reported by a study in Sweden (43). Nonetheless, the path analysis indicated that knowledge positively influenced attitude and practice, and that attitude positively influenced practice. Therefore, improving knowledge should also improve attitude and practice. The present study revealed that knowledge of novel technologies (such as TOMO therapy, CyberKnife, and PT) was dismal and needed improvement. However, participants' attitudes toward accepting advanced precision RT were high. Knowledge gaps exist regarding HNC, the risk of related malnutrition, gene mutations in HNC, the role of gene mutations in treatments, HNC treatment options, and immunotherapy for HNC. The present study unveiled healthcare providers as the primary source of information for participants, aligning with findings from prior research (44, 45). These results underscore the crucial role of healthcare providers in maintaining present knowledge about HNC and treatment options to educate patients adequately. Therefore, healthcare providers were also a focus of this study; a research from America revealed that non-oncologist physicians at a community hospital exhibited poor knowledge of RT (46). Despite participants reporting limited access to medical information through interactions with relatives and friends, potentially due to feelings of embarrassment when discussing their illness, the role of social support shouldn't be underestimated. Stickel et al. (47) found in a survey of participants' knowledge and attitudes toward cancer peer support programs that approximately half of the participants expressed interest in such programs. They sought to gain insights from peer patients' experiences, practical assistance, and emotional support, bolstering their confidence in treatment.

The present findings have significant implications for clinical practice, particularly in terms of participants education and communication strategies. Despite low knowledge of advanced precision RT, participants exhibited a positive attitude, indicating that the suggestion of healthcare providers is a key factor in treatment acceptance (38, 39). This highlights the importance of clear, effective communication from healthcare providers, especially when explaining complex, novel treatments such as TOMO therapy, CyberKnife, and PT. Incorporating more interactive education sessions and utilizing visual aids or simplified explanations can enhance participants' understanding. Moreover, healthcare providers need to maintain up-to-date knowledge to effectively improve the level of patient's understanding. In China, disparities in healthcare infrastructure and resource distribution are evident. The findings also showed significant differences in knowledge between rural and urban populations, underscoring the necessity of tailored educational interventions (32). For participants with lower incomes, lower education levels, or rural residences, simple, easy-to-understand short videos could be used to convey information about advanced precision RT, as videos have proven effective in improving cancer patients' understanding of best practices (48). For participants with higher incomes, higher education levels, or urban residence, advanced precision RT information can be provided by printing information brochures (49) or developing Internet platforms (50) or mobile apps (51).

This study had several limitations. The results of this study represented the present situation of KAP in advanced precision RT for participants with HNC in one research center. Similar situations may exist in most underdeveloped cities in China, which is a relatively common phenomenon, and should be given attention. To address this, future investigations should include participants from multiple research centers to ensure a more representative opinion reflecting varied cognitive levels, attitudes, and practices toward advanced precision RT. The use of self-reported data also presented limitations, including recall and social desirability biases, which may lead to inaccurate reporting, compounded by the lack of objective verification. The lack of objective verification makes it difficult to confirm the accuracy of the responses, thus requiring careful interpretation of the findings. Moreover, while we collected some socioeconomic indicators, such as residence, education level, and income, these do not fully capture the broader socioeconomic context. Therefore, future studies should address these gaps by incorporating more detailed demographic and socioeconomic data.

In conclusion, participants with HNC have a common lack of knowledge on advanced precision RT, but they have a positive attitude and a proactive practice. Therefore, there is a pressing need for tailored educational and communication initiatives, in order to enhance the development of advanced precision RT in HNC. Furthermore, wider adoption of advanced precision RT technology would improve the efficacy and quality of life for patients with HNC.

## Data Availability

The raw data supporting the conclusions of this article will be made available by the authors, without undue reservation.
