# Supplementary material for: Knowledge, attitude, and practice toward advanced precision radiotherapy among patients with head and neck cancer
Source: Front Public Health. 2024 Oct 17;12:1461808. doi: 10.3389/fpubh.2024.1461808 (PMC11524847; doi:10.3389/fpubh.2024.1461808)
Supplement: Supplementary file 1 [file Table_1.docx]

**Supplementary Table S1.** Attitude dimension distribution

| **Variables** | Strongly agree  n (%) | Agree  n (%) | Neutral  n (%) |
| --- | --- | --- | --- |
| 1. I trust my doctor completely and am willing to communicate promptly with them about my condition and treatment plan. | 361 (82.8) | 72 (16.51) | 3 (0.69) |
| 1. If recommended by my doctor, I am willing to undergo genetic sequencing tests. | 73 (16.74) | 305 (69.95) | 57 (13.07) |
| 1. I believe that radiotherapy has some effectiveness, and if recommended by my doctor, I am willing to try it. | 316 (72.48) | 108 (24.77) | 12 (2.75) |
| 1. I am willing to try targeted drugs such as cetuximab and nivolumab under the recommendation of my doctor. | 77 (17.66) | 311 (71.33) | 46 (10.55) |
| 1. I believe that quitting smoking and alcohol can effectively reduce the occurrence of treatment complications. | 339 (77.75) | 88 (20.18) | 7 (1.61) |
| 1. I believe that strict maintenance of oral hygiene is crucial and can effectively prevent the occurrence of treatment-related oral complications. | 298 (68.35) | 136 (31.19) | 2 (0.46) |
| 1. I believe that maintaining adequate nutrition during treatment is extremely important. | 250 (57.34) | 186 (42.66) | 0 |
| 1. I am willing to learn more about nutritional supplementation during treatment. | 236 (54.13) | 195 (44.72) | 4 (0.92) |
