# Supplementary material for: Knowledge, attitude, and practice toward advanced precision radiotherapy among patients with head and neck cancer
Source: Front Public Health. 2024 Oct 17;12:1461808. doi: 10.3389/fpubh.2024.1461808 (PMC11524847; doi:10.3389/fpubh.2024.1461808)
Supplement: Supplementary file 2 [file Table_2.docx]

**Supplementary Table S2.** Practice dimension distribution

| **Variables** | Always  n (%) | Often  n (%) | Sometimes  n (%) | Occasionally  n (%) | Never  n (%) |
| --- | --- | --- | --- | --- | --- |
| I closely monitor my condition during treatment, paying attention to any adverse reactions. | 275 (63.07) | 146 (33.49) | 12 (2.75) | 2 (0.46) | 1 (0.23) |
| 1. I undergo regular check-ups during treatment to examine my oral, skin, and nutritional status. | 136 (31.19) | 264 (60.55) | 32 (7.34) | 3 (0.69) | 1 (0.23) |
| 1. I actively consult with doctors regarding dietary nutrition and scientific nutrition. | 106 (24.31) | 109 (25) | 193 (44.27) | 26 (5.96) | 2 (0.46) |
| 1. I maintain a balanced and adequate diet during treatment. | 147 (33.72) | 251 (57.57) | 35 (8.03) | 2 (0.46) | 1 (0.23) |
| 1. I strictly abstain from smoking and alcohol consumption during treatment. | 356 (81.65) | 77 (17.66) | 2 (0.46) | 0 | 1 (0.23) |
| 1. I actively maintain oral hygiene and drink plenty of water during treatment. | 329 (75.46) | 104 (23.85) | 2 (0.46) | 1 (0.23) | 0 |
